# The Outcomes of App-Based Health Coaching to Improve Dietary Behavior Among Nurses in a Tertiary Hospital: Pilot Intervention Study

**DOI:** 10.2196/36811

**Published:** 2022-07-15

**Authors:** Wei Xiang Lim, Stephanie Fook-Chong, John Wah Lim, Wee Hoe Gan

**Affiliations:** 1 Department of Occupational and Environmental Medicine Singapore General Hospital Singapore Singapore; 2 Programme in Health Services and Systems Research Duke-NUS Medical School Singapore Singapore

**Keywords:** smartphone app, weight loss, dietary behavior, nurse, app, mobile health, mHealth, app-based health, health coaching, diet, dietary choice

## Abstract

**Background:**

At the workplace, health care workers face multiple challenges in maintaining healthy dietary behaviors, which is the major factor behind obesity. A hospital-wide mass health screening exercise showed an increasing trend in the prevalence of obesity and median BMI from 2004 to 2019, as well as a higher crude obesity rate among shift workers.

**Objective:**

We aimed to evaluate the effectiveness of mobile app–based health coaching and incentives for achieving weight loss from better dietary choices among hospital nurses.

**Methods:**

We conducted a pilot study from June 2019 to March 2020, involving the use of a health-coaching app by 145 hospital nurses over 6 months. Weight and BMI were self-reported, and food scores were calculated. Data among overweight nurses, shift work nurses, and incentive groups were analyzed.

**Results:**

A total of 61 nurses were included in the final analysis. Of these 61 nurses, 38 (62%) lost weight. The median percentage weight loss was 1.2% (IQR 0%-2.9%; *P*<.001), and the median decrease in BMI was 0.35 (IQR −0.15 to 0.82; *P*<.001), but they were not clinically significant. The median improvement in the food score was 0.4 (IQR 0-0.8). There was no difference between the incentive and nonincentive groups. A total of 49 (34%) participants engaged for ≥8 weeks.

**Conclusions:**

The study demonstrated an association between the use of app-based health coaching and the attainment of some weight loss in nurses, without a significant improvement in the food score. Incentives may nudge on-boarding, but do not sustain engagement.

## Introduction

### Background

Overweight and obesity are the leading risk factors for a multitude of noncommunicable diseases around the world, including cardiovascular diseases, diabetes mellitus, and some types of cancers [1-3]. Health care professionals may themselves be obese [4-6] and less healthy compared with the general population [7,8]. Studies have identified dietary behaviors to be the major factors behind obesity, in particular, the consumption of high caloric food rich in fat and sugar [7,9]. At the workplace, health care workers face multiple challenges in maintaining a healthy diet [6,10,11].

Prior to this study, we conducted a mass health screening exercise for 5171 of our hospital staff members across various job classifications, and the results showed an increasing trend in the prevalence of obesity and median BMI from 2004 to 2019. The median BMI increased from 22.6 kg/m^2^ in 2004 to 23.5 kg/m^2^ in 2019, and the crude obesity rate (BMI ≥30 kg/m^2^) has correspondingly increased from 7.5% to 13.2%.

Comparing the results to the most recently available data provided by Singapore’s National Population Health Survey in 2020 [12], our hospital staff members have a higher crude obesity rate of 12.0% (gender standardized) as compared to a rate of 10.5% in the Singapore population. Hence, we identified the health care worker population as a group increasingly at risk for obesity and other long-term effects associated with obesity.

Among our staff, the crude obesity rate (BMI ≥30 kg/m^2^) among shift workers is higher than that among nonshift workers (13% vs 11%). The barriers to healthy dietary behavior include shift duties, having irregular meals, or absence of a diet plan as a result of shift work [11,13,14]. Standard dietary interventions often involve face-to-face programs during day time working hours and rarely address those performing shift work. Nurses form the majority of the hospital workforce, and they are susceptible to the negative effects of shift duties on nutrition patterns and obesity [15,16].

A systematic review of 136 lifestyle health promotion intervention studies for nurses found that interventions targeting diet, body composition, physical activity, or stress are most likely to have positive outcomes for nurses’ health or well-being [17]. The review also suggested that interventions aimed at improving nutrition amongst nurses commonly result in improved outcomes, especially when the interventions are education based. However, the use of web technology was mainly limited to education websites and not mobile apps. A study involving female health care workers found that a web-based educational tool was not useful for improving and modifying their lifestyle [18].

There have been efforts launched to address obesity and other health outcomes among the general and patient populations using smart apps both locally in Singapore [19-21] and internationally [22-26]. Mobile app–based health coaching has been used in a local study to target the diabetic patient population with a similar study period of 6 months for the participants [19]. Another local pilot randomized controlled trial targeting overweight pregnant women [20] had a different objective to control gestational weight gain and macronutrient intake rather than weight loss. A local randomized controlled trial protocol has also been proposed locally to evaluate the effectiveness of a comprehensive diabetes management package involving 2 health apps over 24 months, with weight loss as its secondary objective [21]. An American study also indicated that the use of diet/nutrition apps is associated with diet-related behavior change, namely increased actual goal setting to eat a healthy diet, increased frequency, and consistency of eating healthy foods [27].

However, based on published literature, there are limited programs using smart apps targeted at health care workers. Therefore, we hope to provide a multi-domain nonface-to-face dietary behavior coaching program by leveraging digital technology. Instead of a prescribed date and time of a physical session, this modality enables nurses to interact and participate in the program any time at their convenience.

### Objective

With funding from Singapore’s Health Promotion Board (HPB), a small pilot study was implemented by onboarding nurses onto a digital app to participate in a dietary behavior health coaching program.

The primary aim of this study was to evaluate the effectiveness of a mobile app for improving dietary behaviors among nurses, especially among shift workers, which include improving quality of food choices and achieving weight loss from better dietary choices.

The secondary aim was to evaluate the effectiveness of incentives for modifying and sustaining health behavioral change.

The data received were analyzed for program effectiveness and for the institution to gain insights into the implementation challenges around the use of digital technology for this purpose. These learning points include participant recruitment strategies, potential problem areas, and real-world operational concerns, all of which will allow us to recommend changes if the program is to be scaled to a much larger population in the future.

## Methods

### Study Design

This was a pilot study conducted within a tertiary acute hospital in Singapore between June 2019 and March 2020. As part of the corporate health improvement initiative and as an extension of the Workplace Alliance for Health Scheme, the hospital’s Department of Occupational and Environmental Medicine partnered the national HPB to start a program of using a commercially available mobile app–based intervention to promote and improve dietary behavior among nurses.

The mobile app is a proprietary healthy lifestyle management program that was originally developed by a private company for use in Singapore for adults with type 2 diabetes mellitus (T2DM) [28]. Screenshots of the mobile app are shown in Multimedia Appendix 1 and Multimedia Appendix 2. It is a commercially available app downloadable from either the Apple App Store or Google Play Store, and users can access its features through paid subscriptions. It comprises the following 2 components: (1) a comprehensive T2DM educational curriculum delivered through online lessons and (2) the mobile app with a health coaching feature. The mobile app enables users to log and monitor their blood glucose levels, weight, meals, and physical activity, which is captured via the mobile phone’s built-in pedometer. The app also serves as a vehicle for accredited dietitians, known as health coaches, to provide personalized feedback to participants on their progress and to present opportunities for improvement.

Based on the quantum of funding secured, we were able to use only part of the suite of features offered by the mobile app, such as weight and meal logs, real-time health coaching, and general health information, for up to 145 users.

### Participants

We recruited 145 nurses who are currently working in the hospital. The recruitment onboarding process took place across 3 months as planned. It was an open recruitment process where instructions for registration were cascaded through nursing leaders. Registration was on a first-come first-serve basis. Participants were included if they (1) were nurses within the hospital and (2) had a smart mobile device. We did not specifically set a target proportion of shift workers, or overweight or obese workers. Participants were excluded if they (1) were not permanent staff of the hospital or (2) did not have a smart mobile device. All successfully registered participants received 6 months of free unlimited access to mobile app–based health coaching. The app allowed them to (1) voluntarily log their weight and food intake via photos or text comments, (2) communicate with a dedicated health coach in real time via the secured in-app messaging feature, and (3) receive general health information provided within the app, such as food choices during Ramadan, the fasting month observed by Muslims.

The participants were also randomized to the low incentive group (group A) or the high incentive group (group B) in a 73:72 allocation ratio. Both groups of participants were provided with full access to the mobile app–based health coaching and its services for 6 months. Group A received S $15 (US $10.70) worth of health points in HPB’s Healthy 365 app at the end of the first 3 months and no further incentive for the next 3 months, while group B received S $30 (US $21.40) worth of health points in HPB’s Healthy 365 app at the end of 6 months. Healthy 365 is a mobile app by Singapore’s HPB, which aims to encourage users to adopt a healthier lifestyle through the use of gamification and rewards [29]. The app seamlessly pairs with fitness tracking devices to help users log their daily step count and amount of time spent on active exercise. Users can sign up for in-app challenges and health programs to earn health points, which they can redeem for healthy lifestyle rewards from a catalogue. The criterion for the group A incentive was engagement with the app for 8 weeks out of the first 12 weeks of the program. The criteria for the group B incentive were (1) at least 4 engaged weeks in the last 12 weeks of the program and (2) qualification for the first incentive.

### Data

Data, such as biodata, age, height, and weight, were self-reported by the participants in the app, and BMI was calculated based on self-reported height and weight. Food scores were assigned by the mobile app’s dieticians and nutritionists based on the user’s uploaded photos or text comments. The meals were given a rating of 1 to 5, in half-point increments, with 1 being the least healthy and 5 being the healthiest. The food log meal reading improvement was then derived by subtracting the mean of the last 5 food logs from the mean of the first 5 food logs. The health coaches also trended the frequency of engagement and reached out to participants who had reduced engagement with the app. An engaged week was defined as the user logging at least one food, weight, or message into the app. However, biochemical data, such as fasting glucose and lipid profiles, were not collected.

All data were self-entered by program participants into the app itself. Data from the app were kept strictly confidential and safe on a secured password-protected server, with limited access given to program coaches. Access to data was limited to the program team only. The hospital team obtained only deidentified data for program evaluation. No identifiable data were used for analysis.

### Ethics Approval

We conducted the study according to the tenets of the Declaration of Helsinki and obtained approval from the SingHealth Centralized Institutional Review Board (CIRB 2019/2456). Consent was embedded in the mobile app, as part of the on-boarding/registration process to create an account within the app. Potential candidates were considered to be enrolled in the program only after they pressed the accept button on the consent page.

### Statistical Analysis

Data for continuous variables were reported as median (IQR), as most data had a skewed distribution, and categorical variables were reported as count (%). Comparisons of categorical data between comparison groups of overweight nurses, shift workers, and randomization arms were performed using the chi-squared test, and the Mann-Whitney test was used for continuous variables. All tests were 2-sided, with a *P* value <.05 taken as indicating statistical significance. Statistical analyses were performed using IBM SPSS Statistics Version 19 software (IBM Corp).

## Results

A total of 145 hospital nurses were enrolled in the program. Table 1 shows the demographic characteristics of our study population. There were 129 (89.0%) females and 16 (11.0%) males. The median age was 34 years (IQR 30-43 years, range 22-66 years). The majority of nurses were Chinese (67/145, 46.2%) and Malay (48/145, 33.1%). Moreover, 114 (78.6%) nurses were overweight (BMI ≥23 kg/m^2^). The median starting weight was 67 kg (IQR 57-78 kg, range 38-125 kg), and the median starting BMI was 26.0 kg/m^2^ (IQR 23.0-30.0 kg/m^2^, range 17.0-45.0 kg/m^2^). Overall, 85 (58.6%) nurses were shift workers.

Among the 145 nurses recruited for the program, 49 (33.8%) engaged for ≥8 weeks and 38 (26.2%) engaged for ≥12 weeks. The median total logs (meal and weight logs) was 17 (IQR 4-37), and the median total touchpoints (Ask Coach) was 47 (IQR 17-104) per participant. Among the 61 nurses in the final analysis, the median interval between weight logs was 10 weeks (IQR 5-21 weeks).

The results of 61 (42.1%) participants were included in the final analysis (Table 2). The other 84 participants were excluded as they did not self-report their before and after weights. The median weight loss was 0.8 kg (IQR 0-2.3 kg; *P*<.001), and the median percentage weight loss was 1.2% (IQR 0%-2.9%; *P*<.001). Of the 61 participants, 38 (62.3%) lost weight. There was a median decrease in BMI of 0.35 (IQR −0.15 to 0.82; *P*<.001). The median improvement in the food score was 0.4 (IQR 0-0.8; *P*=.07). Among the 84 participants who were excluded due to insufficient weight data, 16 had sufficient food logs for analysis. When analyzed together with the group of 61 participants having weight data, the median improvement in the food score (77 participants with food score data) was 0.4 (IQR −0.1 to 0.8; *P*=.07).

**Table 1:** Demographic characteristics of the participants.

Characteristic		Value (N=145)
**Gender, n (%)**		
	Female	129 (89.0)
	Male	16 (11.0)
**Ethnicity, n (%)**		
	Chinese	67 (46.2)
	Indian	14 (9.7)
	Malay	48 (33.1)
	Others	16 (11.0)
Age (years), median (IQR)		34 (30-43)
Height (m), median (IQR)		1.59 (1.56-1.63)
Starting weight (kg), median (IQR)		67 (57-78)
Starting BMI (kg/m^2^), median (IQR)		26 (23-30)
**Shift work, n (%)**		
	Nonshift	60 (41.4)
	Shift	85 (58.6)

**Table 2:** Analysis of the health outcomes of participants.

Outcome	Overall (n=61), median (IQR)	*P* value	Overweight or obese (n=49), median (IQR)	Nonoverweight or nonobese (n=12), median (IQR)	*P* value	Shift work (n=33), median (IQR)	Nonshift work (n=28), median (IQR)	*P* value	Double incentive (n=30), median (IQR)	Single incentive (n=31), median (IQR)	*P* value
Weight loss (kg)	0.8 (0 to 2.3)	<.001	N/A^a^	N/A		N/A	N/A		N/A	N/A	
Percentage weight loss	1.2 (0 to 2.9)	<.001	1.2 (−0.2 to 3.1)	0.5 (0 to 1.9)	.60	1.6 (0 to 3.6)	0.4 (−0.1 to 2)	.12	1.1 (−0.2 to 1.0)	1.2 (0 to 1.0)	.53
Reduction in BMI	0.35 (−0.15 to 0.82)	<.001	0.41 (−0.14 to 0.9)	0.24 (−0.11 to 0.57)	.55	0.43 (−0.8 to 0.96)	0.23 (−0.20 to 0.59)	.22	N/A	N/A	
Improvement in the food score	0.4 (0 to 0.8)	.07	0.5 (0.1 to 0.9)	0.2 (−0.1 to 0.6)	.13	0.3 (−0.6 to 1.4)	0.4 (−0.6 to 1.3)	.67	0.4 (−0.1 to 0.8)	0.4 (0.1 to 0.8)	.83

^a^N/A: not applicable.

Among the 61 participants, there were 49 (80.3%) overweight or obese nurses (BMI ≥23 kg/m^2^), and we observed that 63.3% (31/49) of them lost weight compared to 58.3% (7/12) of nonoverweight or nonobese nurses. The median percentage weight loss in the overweight or obese group was 1.2% (IQR −0.2% to 3.1%), and the value was higher compared to 0.5% (IQR 0%-1.9%) in the nonoverweight or nonobese group. There was a greater reduction in BMI in overweight or obese nurses (0.41, IQR −0.14 to 0.9) compared to that in nonoverweight or nonobese nurses (0.24, IQR −0.11 to 0.57). The overweight or obese group also showed better improvement in the food score, with a median improvement of 0.5 (IQR 0.1-0.9) compared to 0.2 (IQR −0.1 to 0.6) in the nonoverweight or nonobese group. However, both differences were not statistically significant.

Among the 61 participants, there were 33 (54.1%) shift workers. We observed that the median percentage weight loss in shift workers was 1.6% (IQR 0%-3.6%), and the value was higher compared to 0.4% (IQR −0.1% to 2%) in nonshift workers. There was also a greater reduction in BMI in shift workers (0.43, IQR −0.8 to 0.96) than nonshift workers (0.23, IQR −0.20 to 0.59). However, both differences were not statistically significant. There was also no difference in meal improvement between shift workers and nonshift workers.

Comparing group B participants (n=30) who received 2 incentives (3rd month and 6th month) and group A participants (n=31) who received a single incentive (3rd month), there were no differences in both percentage weight loss and improvement in the food score.

## Discussion

### Principal Findings

Smart apps are being increasingly employed to address obesity and other health outcomes among the general population and patient populations. App-based interventions have been shown to be effective in promoting weight control through shaping positive dietary behavioral change, thus leading to desired health outcomes in the long term [30,31]. One main aspect that smart apps focus on is dietary self-monitoring, which is the cornerstone of behavioral weight loss treatment and has been associated with greater weight loss [31-34]. The health coaching mobile app in this pilot study was originally developed for use in Singapore as a proprietary lifestyle management program for adults with T2DM [19].

The WHO has also recommended the use of nonfood rewards in obesity treatments [35]. However, previous trials and systematic reviews have reported mixed results [36-39], with some studies suggesting that financial incentives may be appropriate when properly administered, but behavioral maintenance persists as a weakness in most cases [40,41]. Locally, the National Steps Challenge [42] is an incentive-based national program to encourage Singaporeans to be more physically active, and 90% of those who have already received all rewards continue to persist for at least another 2 months.

The median percentage weight loss was 1.2% for overweight participants after 6 months of app-based health coaching, compared to a 2.3% reduction in baseline weight in a local study by Koot et al [19]. In a systematic literature review by DiFilippo et al [23], 2 out of 3 randomized controlled trials reported percentage weight loss values of 3.2% and 2.7%. These were lower than the minimum clinically meaningful weight loss of 5%, even though the weight loss was statistically significant. The guideline of the National Heart, Lung, and Blood Institute recommends a 10% reduction in weight for overweight and obese people [43], although considerable literature indicates that 5% weight loss is clinically meaningful and associated with reduced health risks [44].

Another meta-analysis of 12 studies by Flores Mateo et al [24] found that those who used a mobile app showed significant decreases in weight of 1.04 kg and BMI of 0.43 kg/m^2^ compared with control groups. This is higher than the median weight loss of 0.8 kg and median decrease in BMI of 0.35 kg/m^2^ in our study. While the meta-analysis included studies with a duration of 6 weeks to 9 months, more than half of the studies had physical activity interventions embedded in the mobile apps. Most of the studies involved only overweight patients, and only 2 of the 12 studies reported high attrition rates. These may explain the higher weight loss and median decrease in BMI in the other studies. Although the median reductions in percentage body weight were modest in our study, it should not be expected to achieve clinically meaningful weight loss with a single weight loss intervention when compared with programs with multiple interventions [45,46]. We recognize that health behavior modification is complex and requires a multi-faceted yet integrated approach. However, it is not the intent of this study to delve into surrounding health behavioral change, such as through the use of the Health Belief Model as an explanatory framework. The mobile app allows fast onboarding and may offer easy access to some of these other multiple interventions that are available in the full version of the app, such as glucose monitoring and physical activity tracking. Further studies can evaluate the effectiveness of an integrated program or workplace health promotion that includes a mobile intervention and its full suite of features.

A systematic review of 12 studies by Wang et al [25] found that the greatest weight loss differences were seen in patients who employed multiple tactics within their weight loss strategy. The authors postulated that while the net effect of the joint nontechnological intervention is unknown, it suggests that even though applications alone promote the self-regulation aspect of weight loss behaviors, they ought to be used in conjunction with other avenues for weight loss. A recent review by Ghelani et al [26] also found that randomized controlled trials have reported smartphone apps to be ineffective for weight management as stand-alone interventions, as they were reported to not achieve the clinically significant target percentage weight loss of 5%. The authors suggested that mobile apps showed potential to be used as an adjunct to other weight management interventions instead.

We observed that engagement levels fell greatly after the short-term engagement period of 3 months, which is consistent with other local studies using smartphone apps [19,20]. This may potentially attenuate the longer-term benefits of sustained lifestyle management, hence accounting for the clinically insignificant weight loss among the studies. The fall in sustained engagement was before Singapore had its first case of COVID-19 [47], and local hospitals had already begun preparatory work and work intensification prior to that. Less than half (61/145, 42.1%) of our participants managed to log their before and after weights. Given the self-reported nature of the dietary and weight data, the accuracy is subjected to social desirability bias and recall bias. Based on systematic reviews performed, high attrition rates are not uncommon in weight-loss intervention programs, and the reasons are varied and complex [48-51]. We also postulate that the low adherence may be due to more considerable effort required to self-monitor diet than to monitor physical activity through a passive method of using motion sensors or pedometers [34,46,52]. We suggest to appoint internal ambassadors to champion the effort and to increase peer-driven engagement, as well as to engage users with in-person events, such as booths providing in-person app support, receiving feedback, and providing healthy snacks as incentives in future studies.

### Strengths and Limitations

The strengths of our study are its prospective study design and the use of standard protocols in dietary food score assessments by qualified dietitians. The limitations of the study include the small sample for analysis relative to the number of enrolled participants and the exclusion of a group of participants due to incomplete self-entered data. As this was a pilot study, the sample size was small in proportion to the nursing population of the hospital, which is around 4000 nurses. Moreover, the use of app features and the frequency of engagement with health coaches were entirely voluntary and up to the individual. Hence, the benefits of dietary modification and extent of weight management were largely dependent on user initiative. The study was also limited by its short duration, and it was not able to demonstrate the longer-term sustainability of health behavioral change and its attendant benefits.

Interestingly, we also observed no differences in percentage weight loss, improvement in food scores, and number of touch points between the low and high incentive groups. However, this can be attributed to the incentives being not too significant in terms of monetary value. According to a systematic review by Purnell et al, larger incentives are associated with better outcomes, although behavior change did not appear to be maintained [36]. Maintenance of behavior could be addressed by using financial incentives to facilitate the desired behavioral outcome rather than to reward the completion of the said behavior, as suggested by Burns et al [53]. To increase the compliance of weight reporting and to reduce the conflict of interest between an incentive and self-reported weight loss of a participant, weight should be taken in-person under supervision.

### Future Directions

Although our study used healthy dietary coaching as the main intervention, physical activities play a significant role in weight loss as well [54-56]. We postulate that enrollment in the program may result in a step up in other active lifestyle measures, such as physical activity, that are not monitored and measured. We hope to look at multi-faceted health-seeking behaviors to better understand our context going forward and to include qualitative methods, such as in-depth interviews and focus group discussions, to better focus on our target population. Future studies could also include deeper insights into the nursing occupation, and could analyze the results while taking into account the working conditions and environment. Such analyses would serve to inform how we may influence health behaviors in the health care worker population, and to adjust health promotion interventions to achieve better outcomes.

### Conclusion

Our study has demonstrated some benefits in achieving weight loss among nurses and has shown some indication of increased benefits among shift workers and overweight participants, even without significant improvements in food scores. We recommend a shorter duration of incentives (3 months) due to a decay in the engagement rate with time. We found that incentives may nudge onboarding, but do not result in sustainment of engagement over time. Future efforts should assess the effectiveness of mobile app–based health promotion using a randomized controlled trial of at least 12 months to evaluate longer-term health behavioral changes and health outcomes. It may also be useful to include more outcome parameters, such as blood pressure, random glucose, and lipid profile. Mobile apps should be used in conjunction with other nontechnological interventions, such as physical activity, group sessions, and face-to-face health coaching, to achieve a more positive effect overall.

## Appendix

Multimedia Appendix 1 Screenshot of the mobile app and in-app health coaching.

Multimedia Appendix 2 Screenshot of the mobile app, weight logging, and food scoring.

## Abbreviations

HPB: Health Promotion Board
T2DM: type 2 diabetes mellitus
