# Supplementary figures and images for: The Outcomes of App-Based Health Coaching to Improve Dietary Behavior Among Nurses in a Tertiary Hospital: Pilot Intervention Study
Source: JMIR Nurs. 2022 Jul 15;5(1):e36811. doi: 10.2196/36811 (PMC9338416; doi:10.2196/36811)

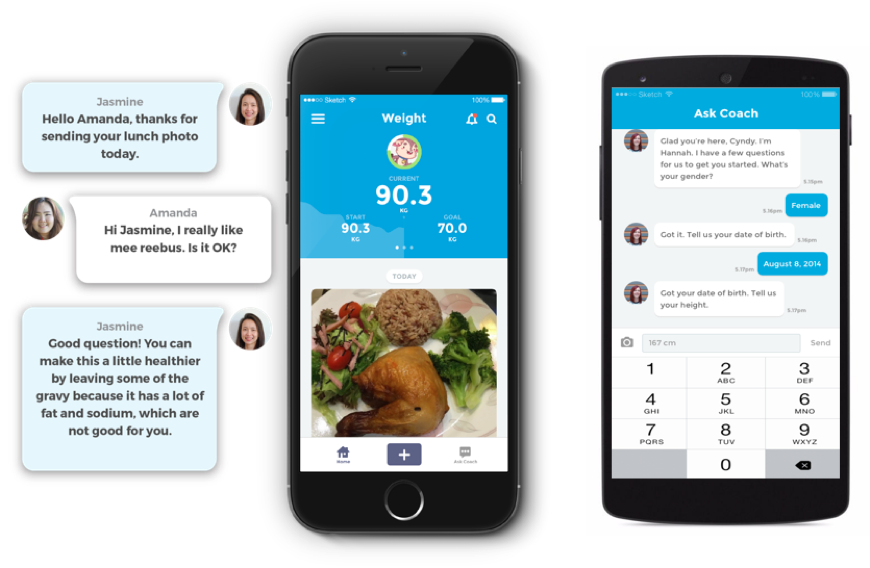

Supplement: Multimedia Appendix 1 [file nursing_v5i1e36811_app1.png]

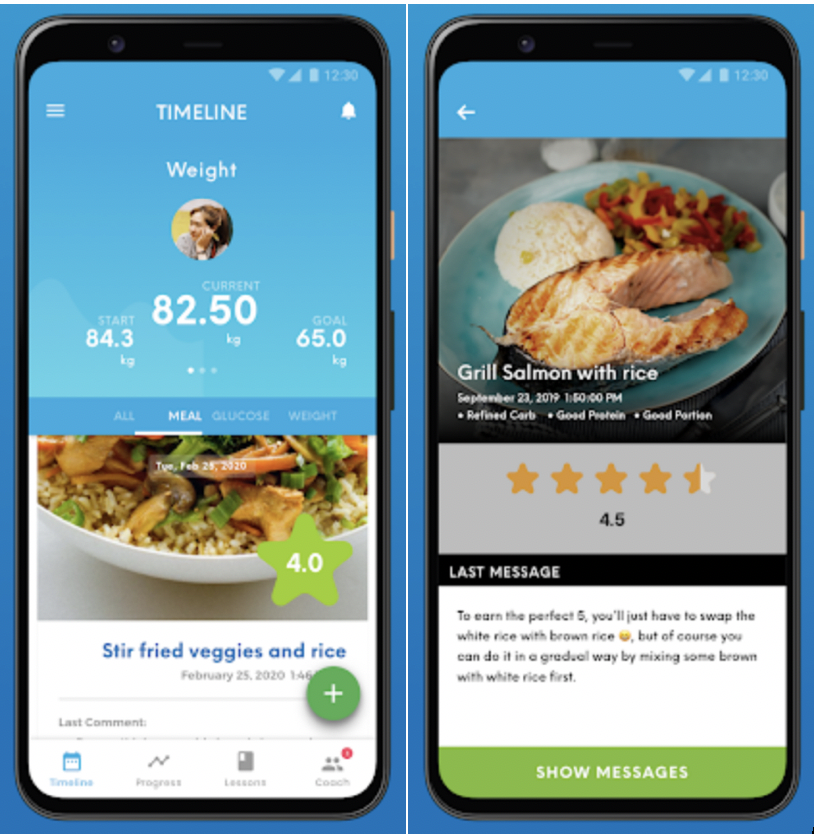

Supplement: Multimedia Appendix 2 [file nursing_v5i1e36811_app2.png]
